# Supplementary material for: DNA methylation signatures of Alzheimer’s disease neuropathology in the cortex are primarily driven by variation in non-neuronal cell-types
Source: Nat Commun. 2022 Sep 24;13:5620. doi: 10.1038/s41467-022-33394-7 (PMC9509387; doi:10.1038/s41467-022-33394-7)
Supplement: Supplementary file 1 — Supplementary Information [file 41467_2022_33394_MOESM1_ESM.pdf]

Supplementary Figures

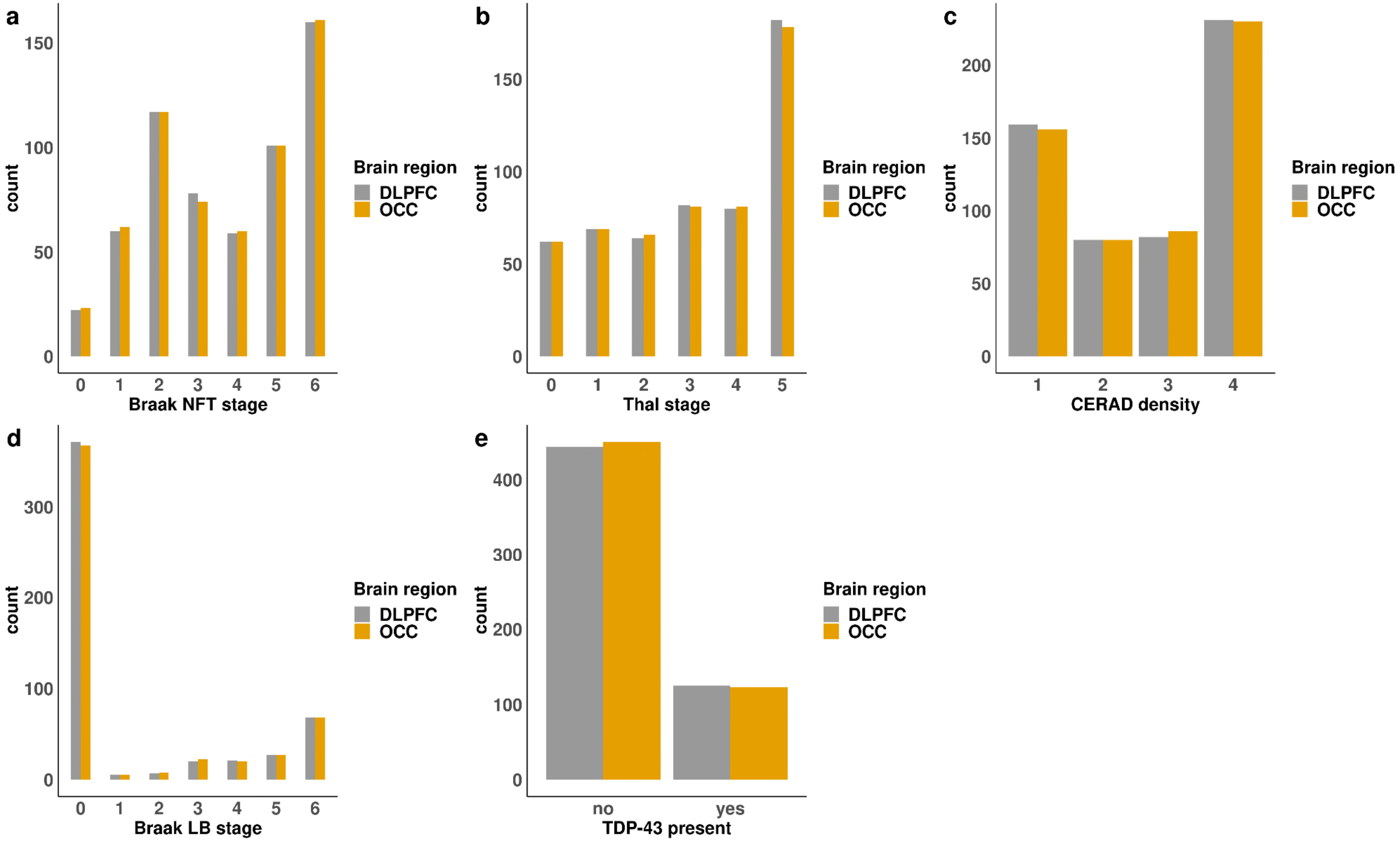

Figure S1: Levels of neuropathology across BDR donors included in this study. Shown is the distribution of neuropathological burden as measured by **a)** Braak NFT stage, **b)** Thal stage, **c)** CERAD density, **d)** Braak LB stage and **e)** TDP43 status.

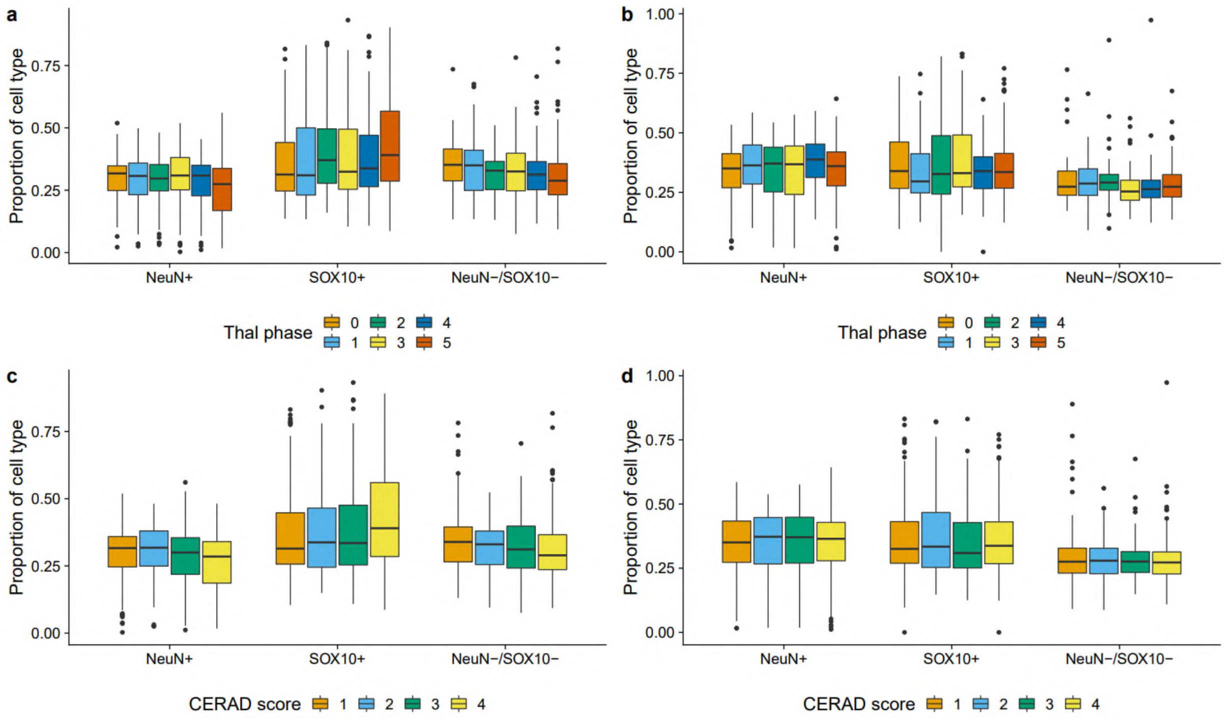

Figure S2: Elevated amyloid pathology is associated with cell proportion estimates derived from DNA methylation data in the DLPFC but not the OCC. Using linear regression models controlling for covariates (see **Methods**) we found that **a)** Thal phase was significantly associated with the proportion of NeuN+ cells (effect size = -1.38, P = 0.006), SOX10+ cells (effect size = 1.26, P = 0.003) and NeuN-/SOX10- cells (effect size = -2.39, P = 0.001) in the DLPFC (N= 539 donors) using cell proportion estimates derived from 'bulk' DNA methylation data. Amyloid pathology is shown on the *x*-axis split by cell-type and estimated cell proportions are shown on the *y*-axis. **b)** In contrast no associations between Thal phase and cell proportion estimates derived from DNA methylation data were observed in the OCC (N= 537 donors, P > 0.008). **c)** CERAD score was significantly associated with the proportion of NeuN+ cells (effect size = -1.38, P = 0.006), SOX10+ cells (effect size = 0.970, P = 0.001) and NeuN-/SOX10- cells (effect size = -1.39, P = 0.004) in the DLPFC (N=552) using neural cell proportion estimates derived from 'bulk' DNA methylation data. **d)** In contrast no associations between CERAD score and cell proportion estimates derived from DNA methylation data were observed in the OCC (N=552 donors, P > 0.008). Shown are boxplots of the estimated proportion of different cell types in groups of donors with different levels of neuropathology, where the middle box represents the interquartile range (IQR), the middle line represents the median and the whisker lines represent the minimum (quartile 1 – 1.5 x IQR) and the maximum (quartile 3 + 1.5 x IQR).

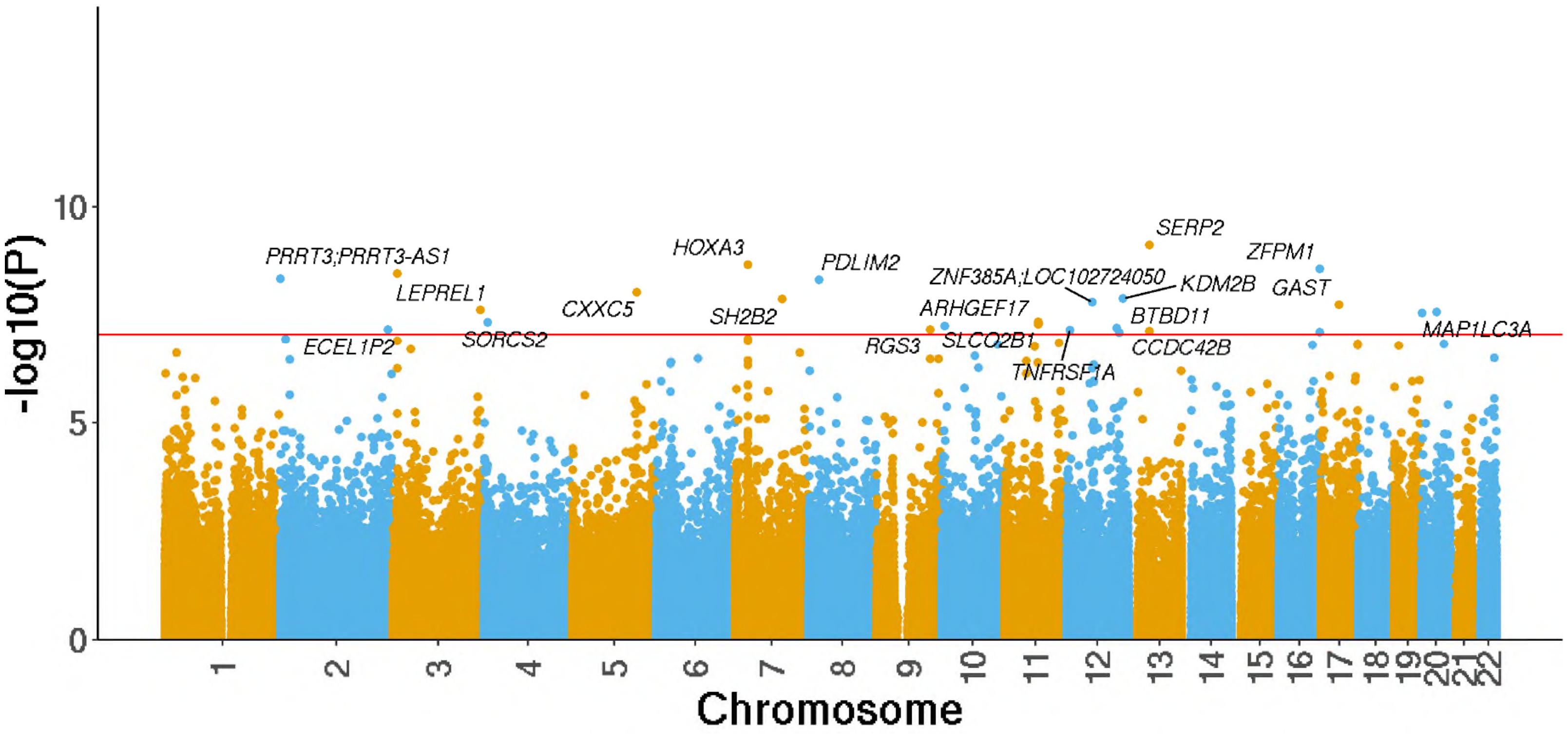

Figure S3: Manhattan plot highlighting cortical DMPs significantly associated with Braak neurofibrillary tangle stage. Linear regressions were run at each DNA methylation site controlling for covariates (N=618 donors). ¶Genes annotated to significant DMPs are labelled. The *x*-axis shows chromosomes 1-22 and the y-axis shows -log10(P), with the horizontal red line representing experiment wide significance (P < 9e-8). A complete list of results is given in **Supplementary Data 3.**

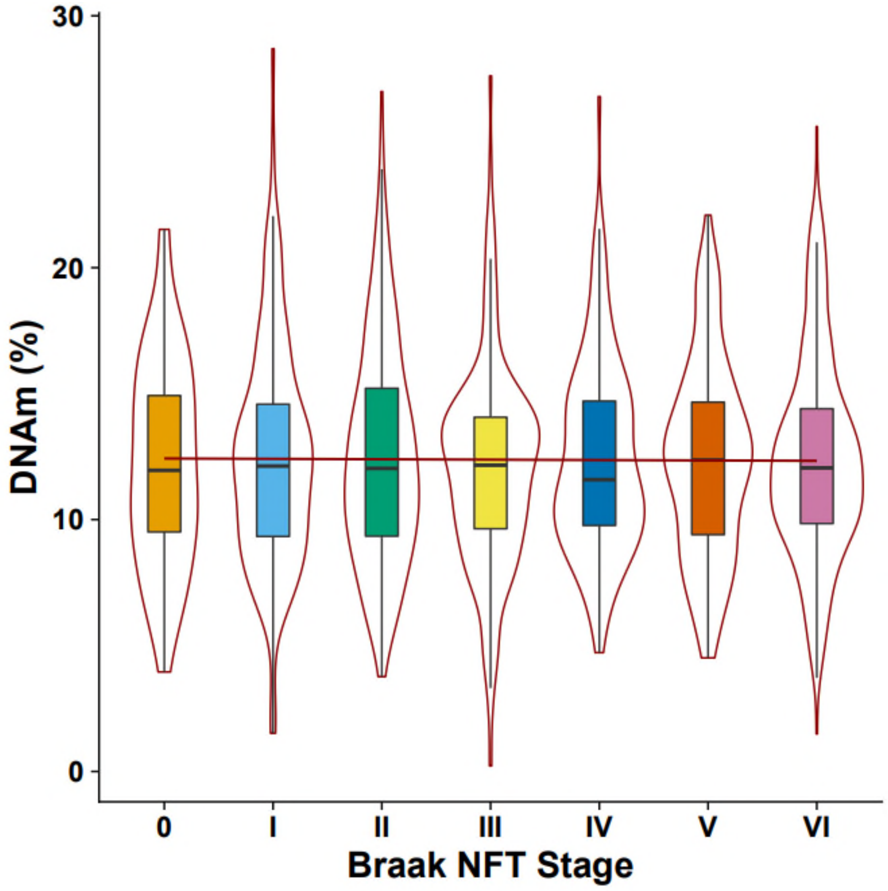

Figure S4: The top-ranked DMP associated with tau pathology. Linear regression identified the most significant DMP associated with Braak NFT stage to be cg16021126 (annotated to *SERP2*), which was significantly hypermethylated with elevated pathology (N = 616, P = 7.48e-10, effect size = 0.286%). Violin plots show DNA methylation values (adjusted for covariates, see **Methods**) across pathology groups, where the box in the middle represents the interquartile range (IQR), the middle line represents the median and the whisker lines represent the minimum (quartile 1 – 1.5 x IQR) and the maximum (quartile 3 + 1.5 x IQR). Pathology stage (Braak NFT Stage) is shown on the *x*-axis and DNA methylation level, adjusted for covariates, is shown on the *y*-axis.

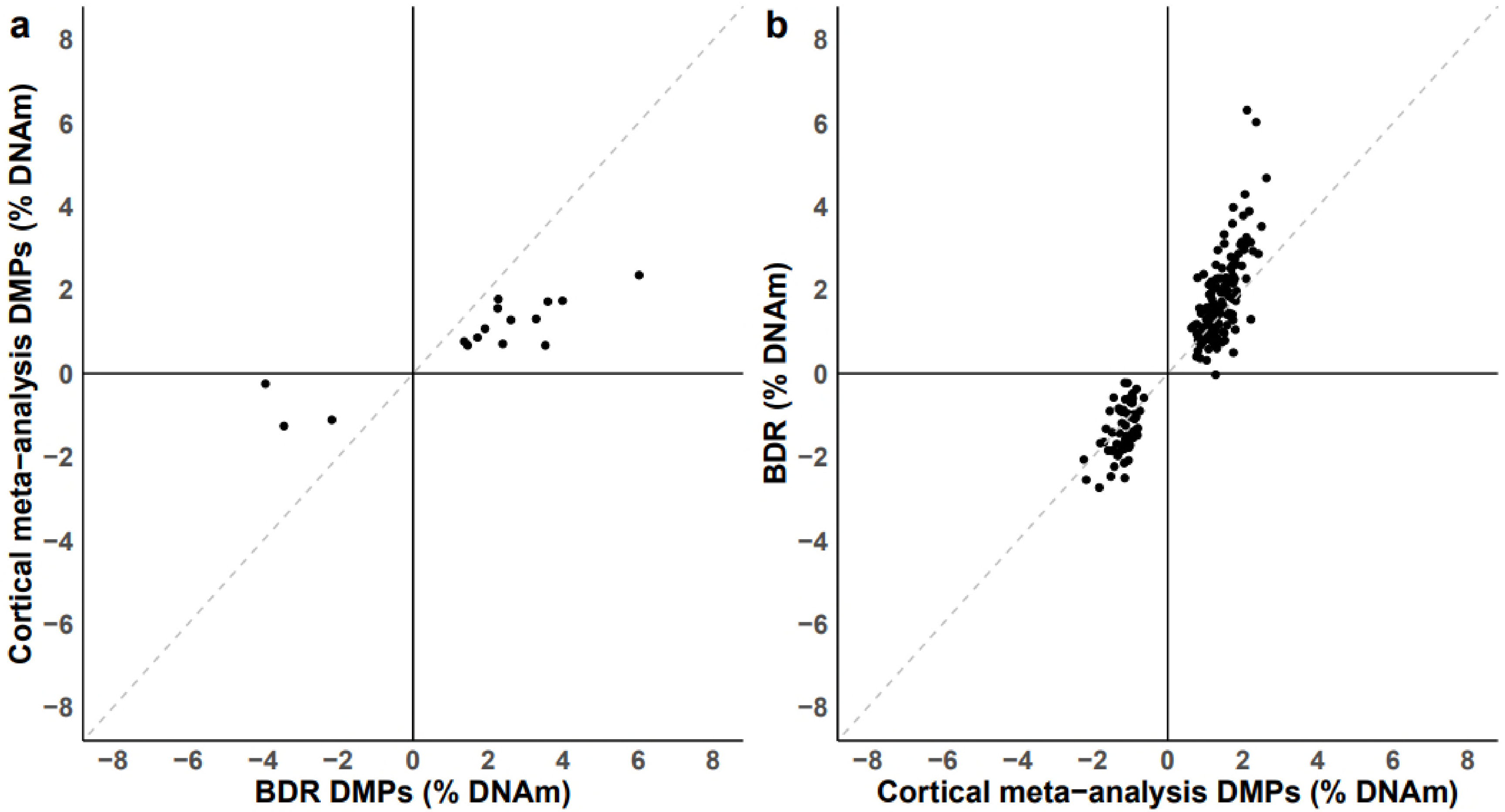

Figure S5: Effects sizes at DNA methylation sites associated with Braak neurofibrillary tangle (NFT) stage in this study are highly consistent with those reported in a recent meta-analysis of Braak NFT stage[13]. Shown for sites tested in both analyses is a comparison of **a)** effect size at Braak NFT-associated DMPs identified in the BDR cohort (n = 16) with those at the same sites in the recent meta-analysis of Braak NFT stage[1] (direction of effect = 100% concordant, sign test P =1.53e-05), and **b)** effect sizes at Braak NFT-associated DMPs identified in the recent meta-analysis (n = 208) with those at the same sites in this study (direction of effect = 100% concordant, sign test P =5.08e-61). The grey dashed line represents y = x.

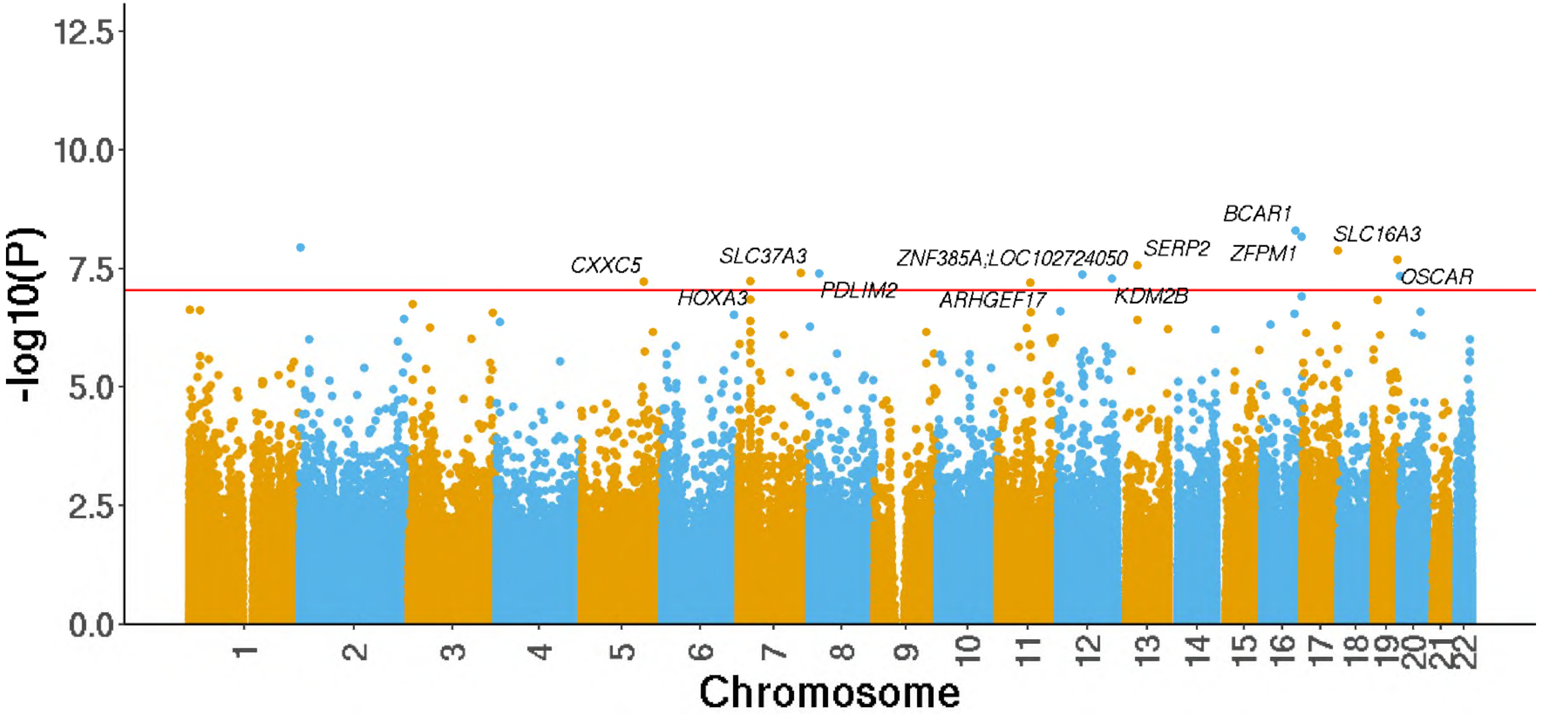

Figure S6: Manhattan plot highlighting significant cortical DMPs associated with the CERAD score measure of amyloid pathology. Linear regressions were run at each site controlling for covariates (N = 572). Genes annotated to significant DMPs are labelled. The *x*-axis shows chromosomes 1-22 and the *y*-axis shows -log10(P), with the horizontal red line representing experiment wide significance (P < 9e-8). A complete list of results is given in **Supplementary Data 3.**

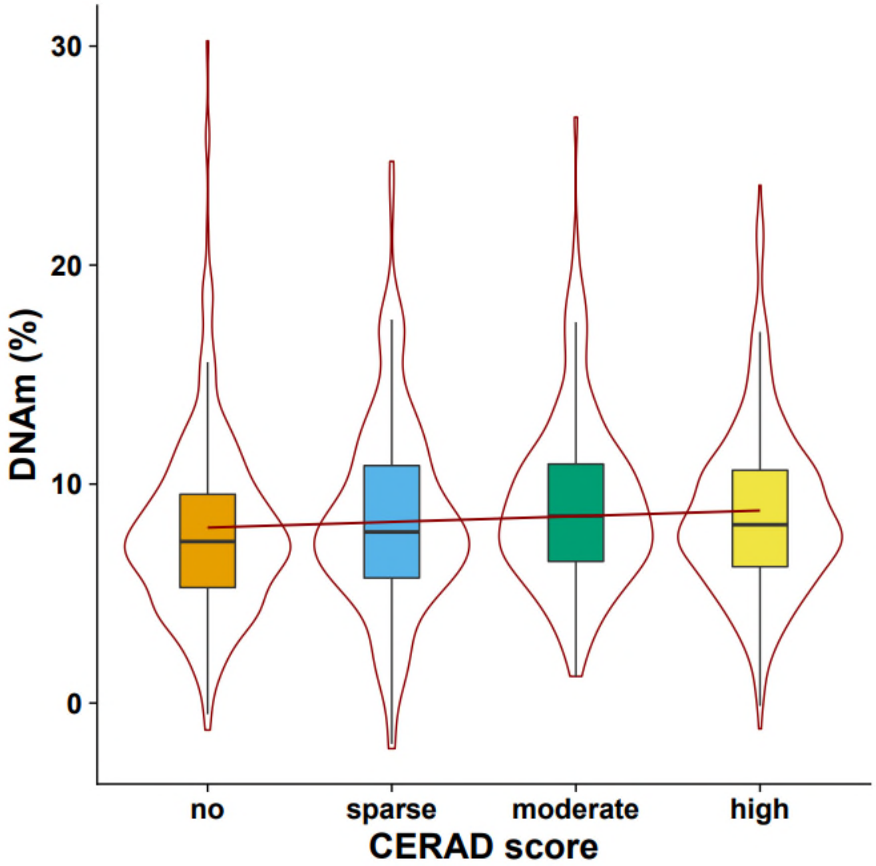

Figure S7: The top-ranked DMP associated with the CERAD score measure of amyloid pathology. Linear regression identified the most significant DMP associated with CERAD score to be cg13515047 (annotated to *BCAR1*), which was significantly hypermethylated with elevated pathology (N = 572, P = 4.96e-09, effect size = 0.442%). Violin plots show DNA methylation values (adjusted for covariates, see **Methods**) across pathology groups, where the box in the middle represents the interquartile range (IQR), the middle line represents the median and the whisker lines represent the minimum (quartile 1 – 1.5 x IQR) and the maximum (quartile 3 + 1.5 x IQR). Pathology stage (CERAD) is shown on the *x*-axis with DNA methylation level, adjusted for covariates, shown on the *y*-axis.

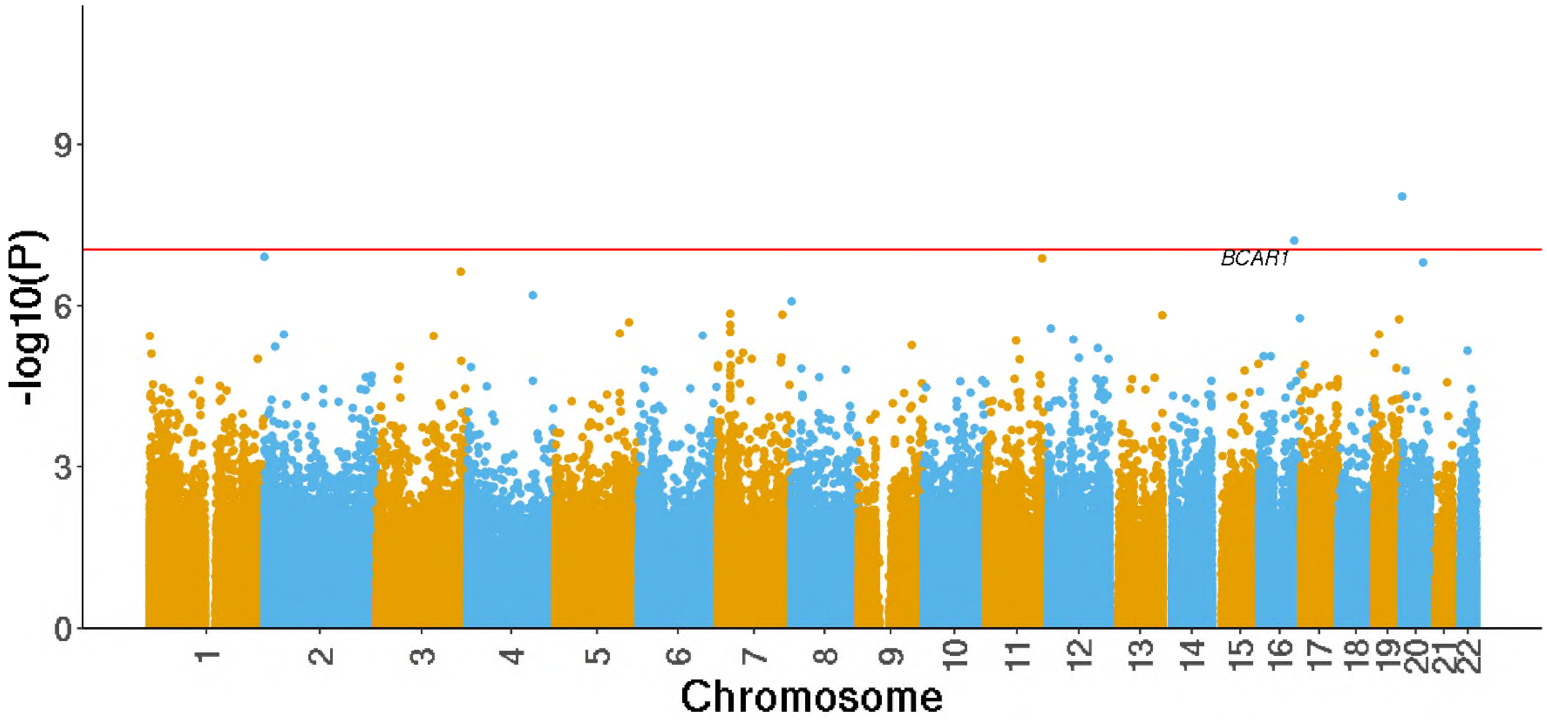

Figure S8: Manhattan plot highlighting significant cortical DMPs associated with Thal Phase measure of amyloid pathology. Linear regressions were run at each site controlling for covariates (N = 558). Genes annotated to significant DMPs are labelled. The *x*-axis shows chromosomes 1-22 and the *y*-axis shows -log10(P), with the horizontal red line representing experiment wide significance (P < 9e-8). A complete list of results is given in **Supplementary Data 3.**

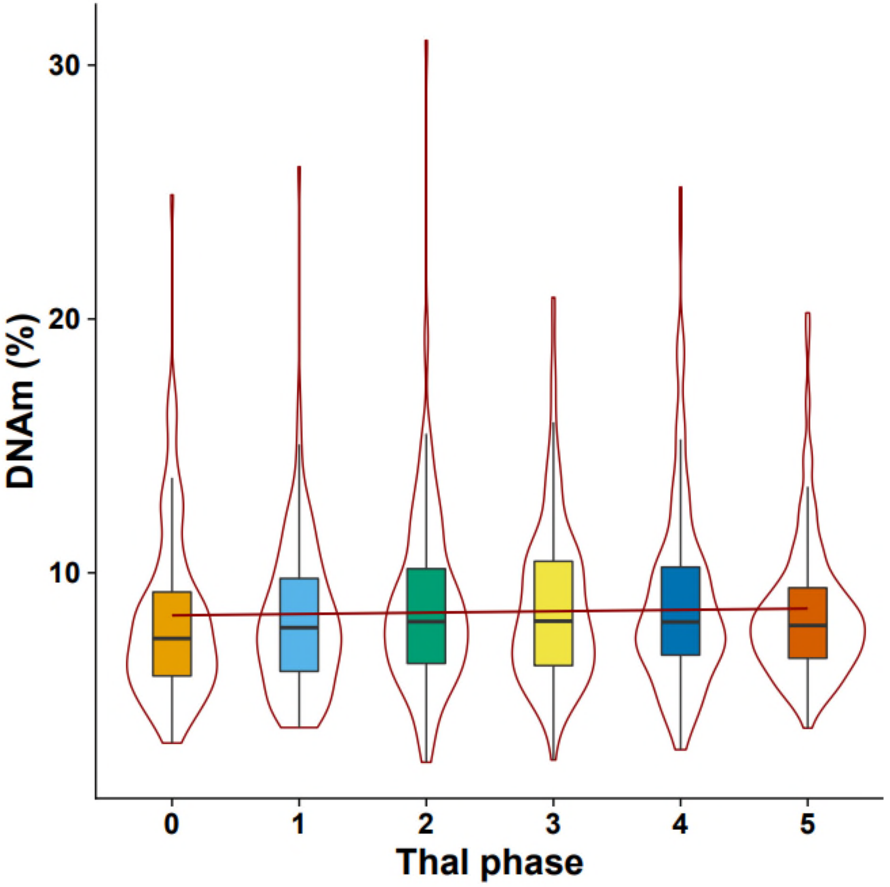

Figure S9: The top-ranked DMP associated with the Thal phase measure of amyloid pathology. Linear regression identified the most significant DMP associated with Thal phase to be cg11658414 (not annotated to a gene), which was significantly hypermethylated with elevated pathology (N = 558, P = 9.11E-09, effect size = 0.299%). Violin plots for the level of DNA methylation (adjusted for covariates, see **Methods**) across pathology groups are shown, where the box in the middle represents the interquartile range (IQR), and the whisker lines represent the minimum (quartile 1 – 1.5 x IQR) and the maximum (quartile 3 + 1.5 x IQR). Pathology stage (Thal) is shown on the *x*-axis with DNA methylation level, adjusted for covariates, shown on the y-axis.

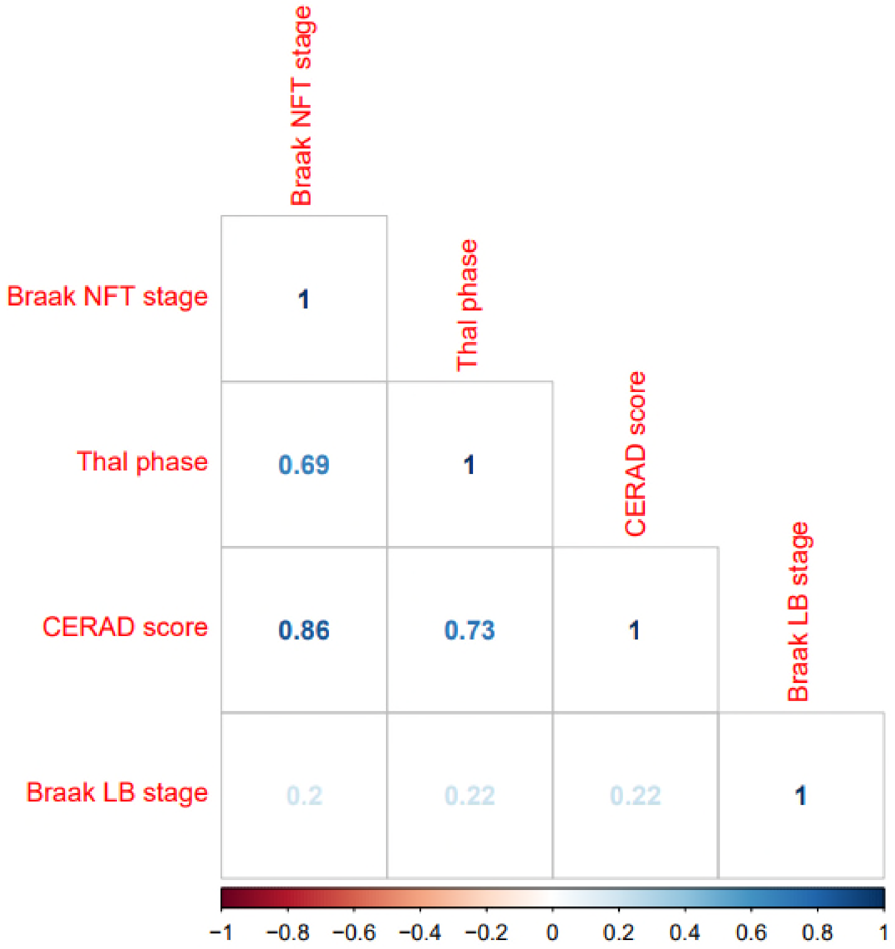

Figure S10: Heatmap showing the strong correlation between different measures of AD neuropathology across BDR donors. There is a weaker correlation between measures of AD pathology and Braak LB stage. NFT = neurofibrillary tangles, LB = Lewy body.

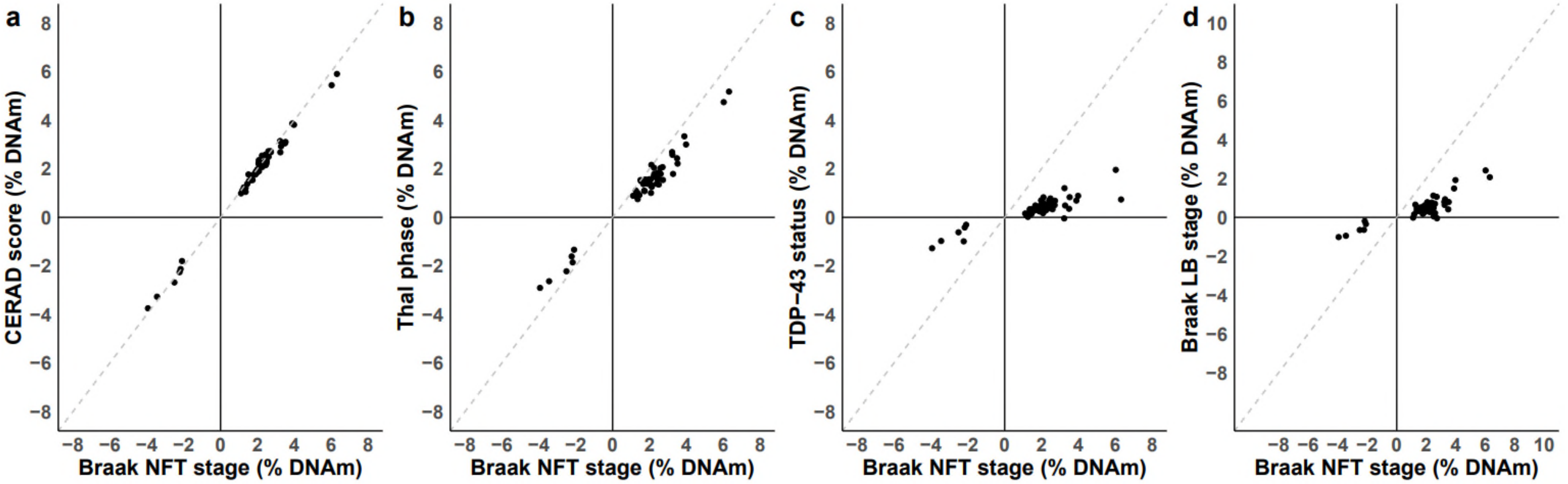

Figure S11: Effects sizes at DNA methylation sites associated with Braak neurofibrillary tangle (NFT) burden are highly consistent with those from EWAS analyses of other dementia neuropathology measures. Shown are the effect sizes for 26 tau-associated DMPs identified in the BDR cohort comparing the results from an EWAS of Braak NFT stage with **a)** CERAD score (direction of effect = 100% concordant, sign test P =1.39e-17), **b)** Thal Phase (direction of effect = 100% concordant, sign test P = 1.39e-17), **c)** TDP-43 status (direction of effect = 98% concordant, sign test P = 7.91e-16) and **d)** Braak LB Stage (direction of effect = 96% concordant, sign test P =2.22e-14). The grey dashed line represents y = x.

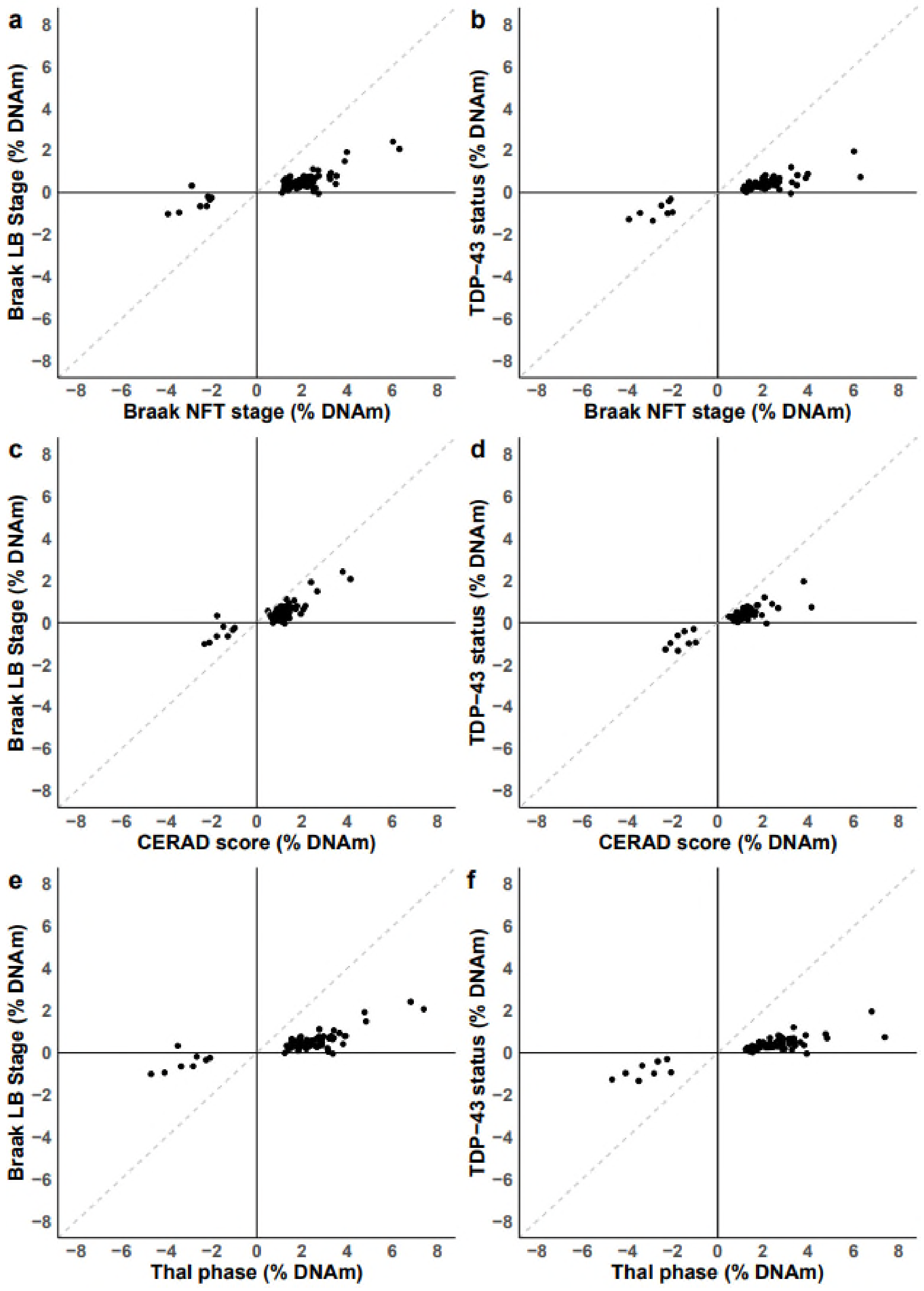

Figure S12: Effects sizes at the 67 cortical DNA methylation sites associated with AD neuropathology are highly consistent across EWAS analyses of individual dementia neuropathology measures. Shown are the effect sizes for the 67 core-AD neuropathology DNA methylation sites from EWAS of the separate neuropathology measures comparing **a)** Braak NFT stage and Braak LB stage (96% concordant; sign test P = 3.4e-16), **b)** Braak NFT stage and TDP-43 status (99% concordant; sign test P = 4.16e-19), **c)** Thal phase and Braak LB Stage (96% concordant; sign test P = 3.4e-16), **d)** Thal phase and TDP-43 status (99% concordant; sign test P = 4.16e-19), **e)** CERAD score and Braak LB Stage (96% concordant; sign test P = 3.4e-16), and **f)** the CERAD score and TDP-43 status EWAS (99% concordant; sign test P = 4.16e-19). The grey dashed line represents y = x.

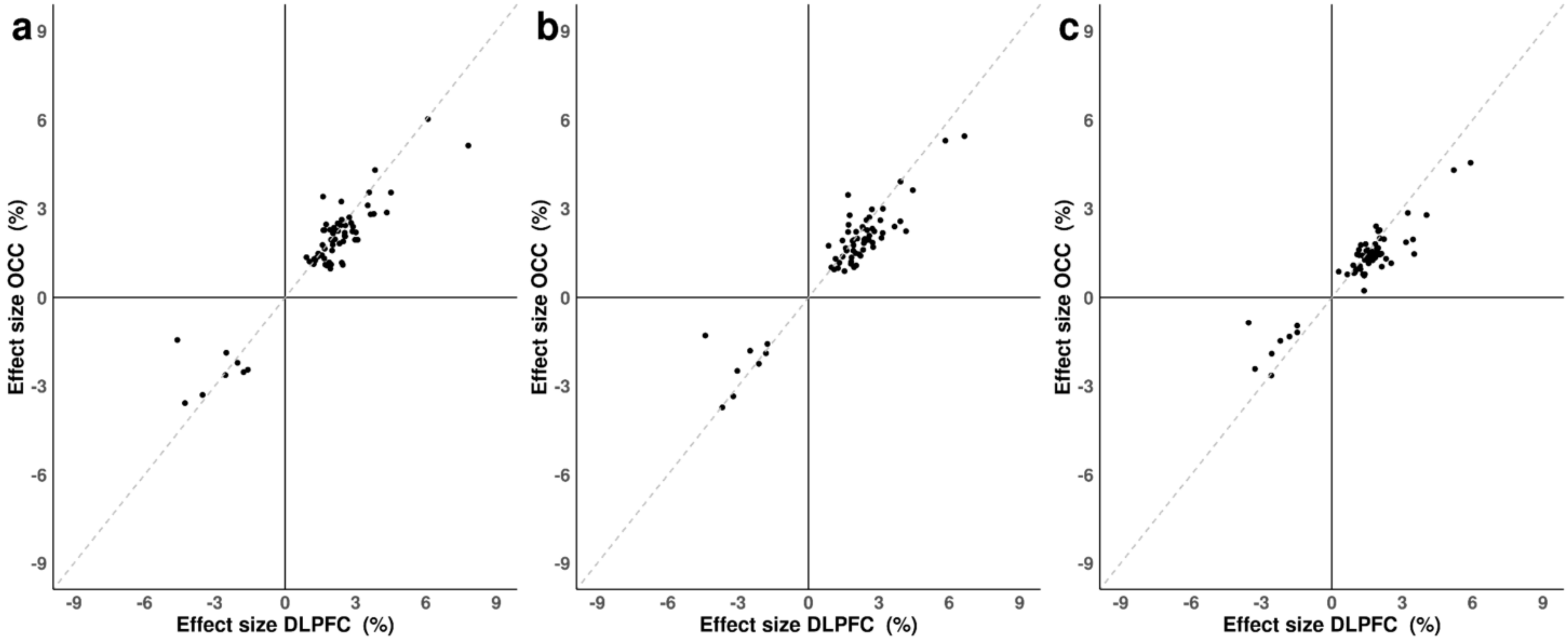

Figure S13: Effects sizes at the 67 cortical DNA methylation sites associated with AD neuropathology are highly consistent across both cortical regions profiled in this study. Shown are the effects sizes for the 67 core AD-associated DNA methylation sites from EWAS of each individual AD neuropathology measure conducted in the DLPFC and OCC separately: **a)** Braak NFT stage (100% concordant; sign test P = 6.78e-21**), b)** CERAD score (100% concordant; sign test P = 6.78e-21) and **c)** Thal phase (100% concordant; sign test P = 6.78e-21). The grey dashed line represents y = x. OCC = occipital cortex; DLPFC = dorsolateral prefrontal cortex.

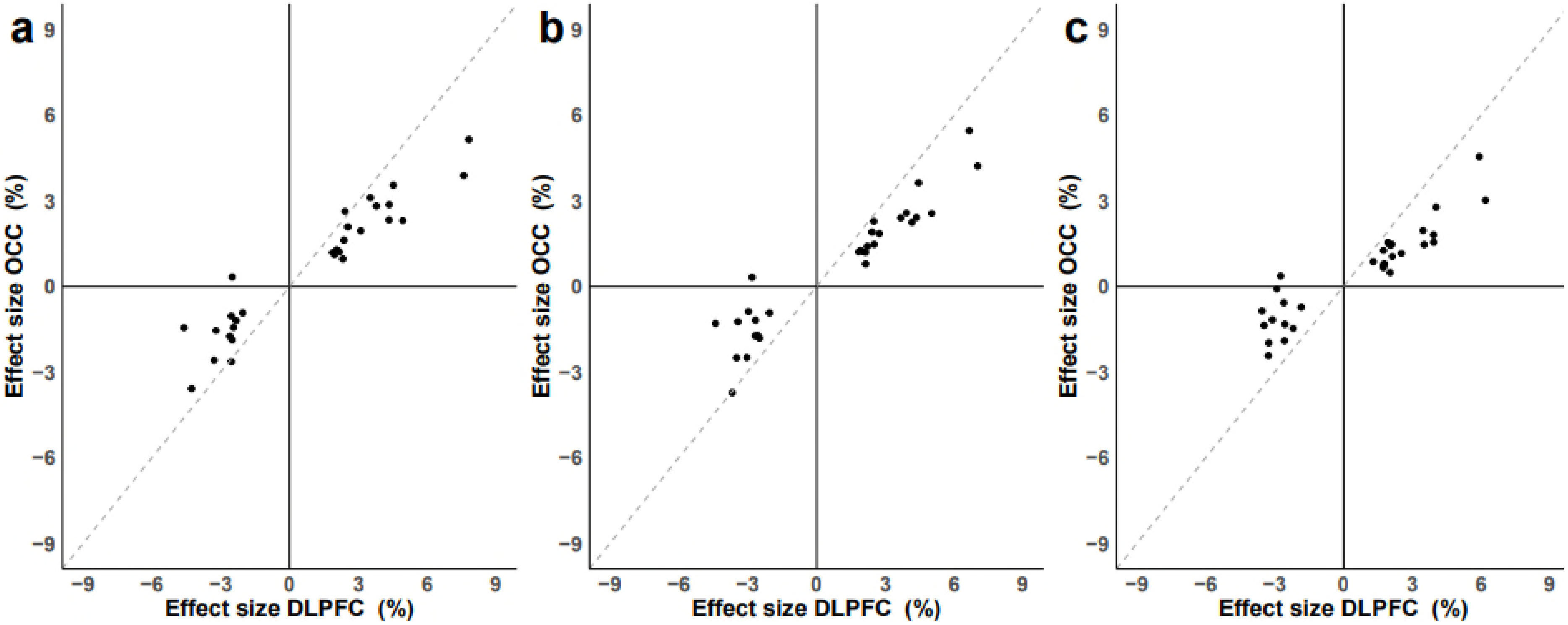

Figure S14: Effect sizes at DNA methylation sites associated with AD neuropathology in the DLPFC are consistent with those identified in the OCC. Shown are the effects sizes for the 30 AD-associated DMPs identified in the DLPFC between the two cortical regions from EWAS analyses of **a)** Braak NFT stage (direction of effect = 97% concordant, sign test P = 2.89e-08), **b)** CERAD score (direction of effect = 97% concordant, sign test P = 2.89e-08) and **c)** Thal phase (direction of effect = 97% concordant, sign test P = 2.89e-08). The grey dashed line represents y = x. OCC = occipital cortex; DLPFC = dorsolateral prefrontal cortex.

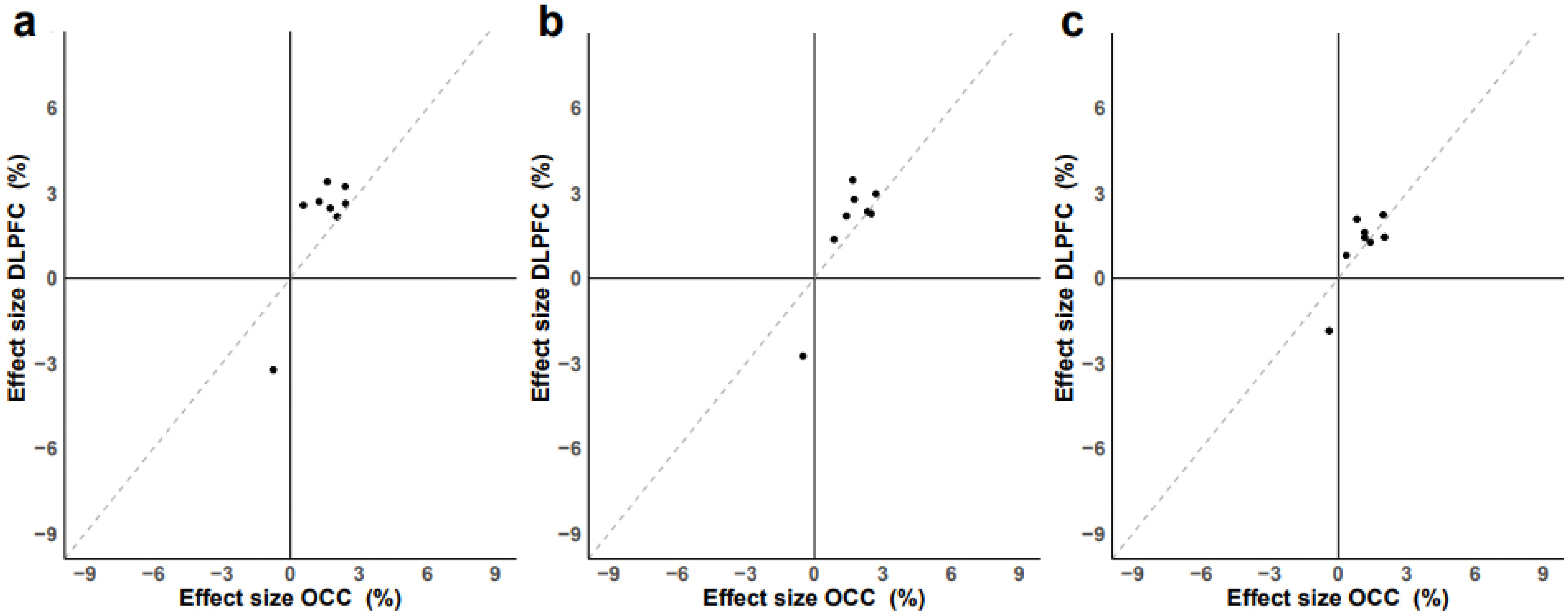

Figure S15: Effect sizes at DNA methylation sites associated with AD neuropathology in the OCC are consistent with those identified in the DLPFC. Shown are the effect sizes at the 8 AD-associated DMPs identified in the OCC between the two cortical regions from EWAS analyses of **a)** Braak NFT stage (direction of effect = 100% concordant, sign test P = 0.00391), **b)** CERAD score (direction of effect = 100% concordant, sign test P = 0.00391), and **c)** Thal phase (direction of effect = 100% concordant, sign test P = 0.00391). The grey dashed line represents y = x. OCC = occipital cortex; DLPFC = dorsolateral prefrontal cortex.

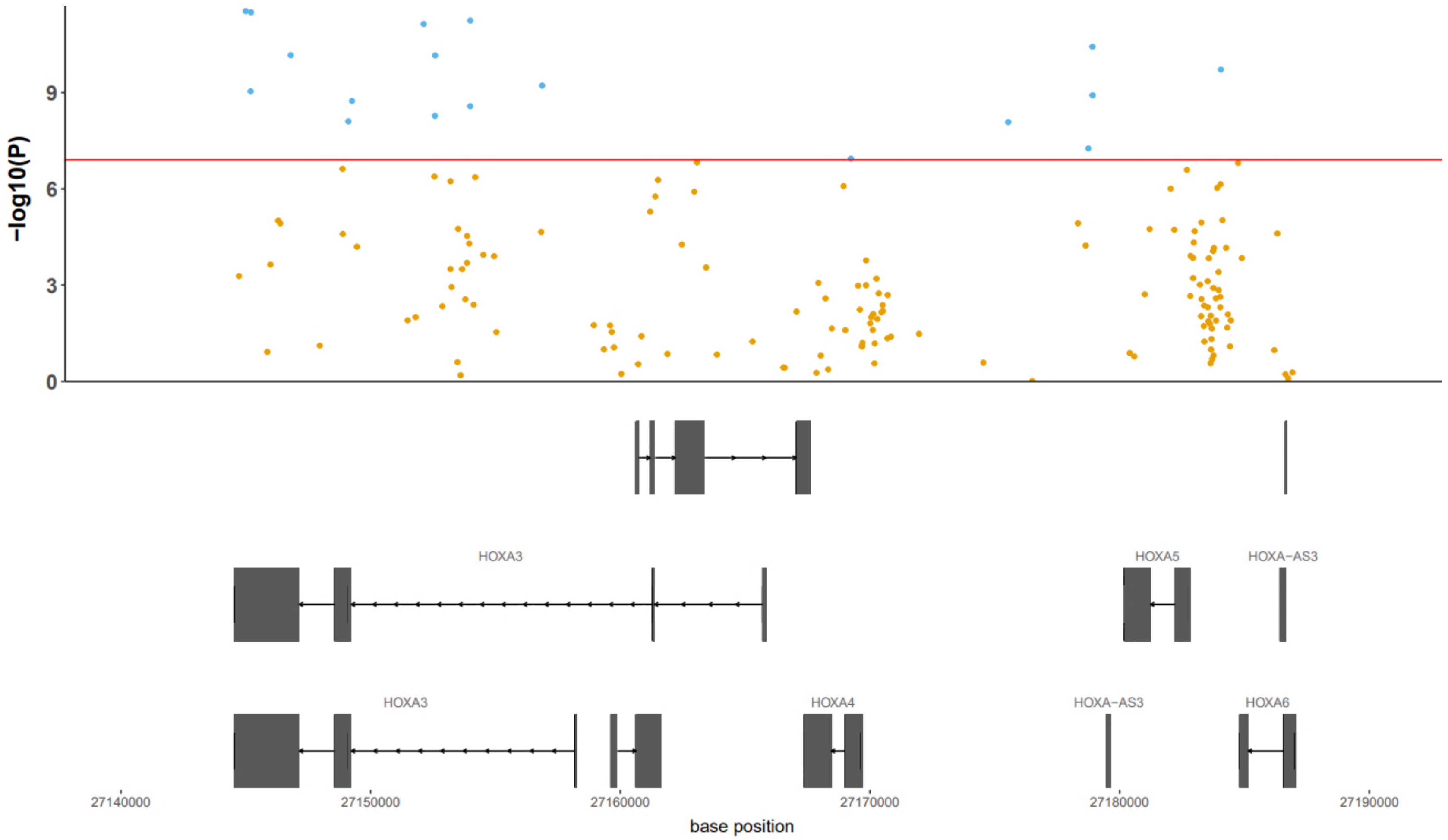

Figure S16: Multiple DMPs in the HOXA gene region are associated with tau pathology. Shown in the top panel is a zoomed-in Manhattan plot spanning the HOXA region on chromosome 7, where the *x*-axis represents genomic location (in bp, hg19), and the *y*-axis represents the –log10 P-value identified by linear regression. Each point on the plot represents an individual DNA methylation site (N = 618). The red horizontal line represents the experiment wide significance threshold (P < 1.24e-07). The bottom track shows the locations of the genes within in this region.

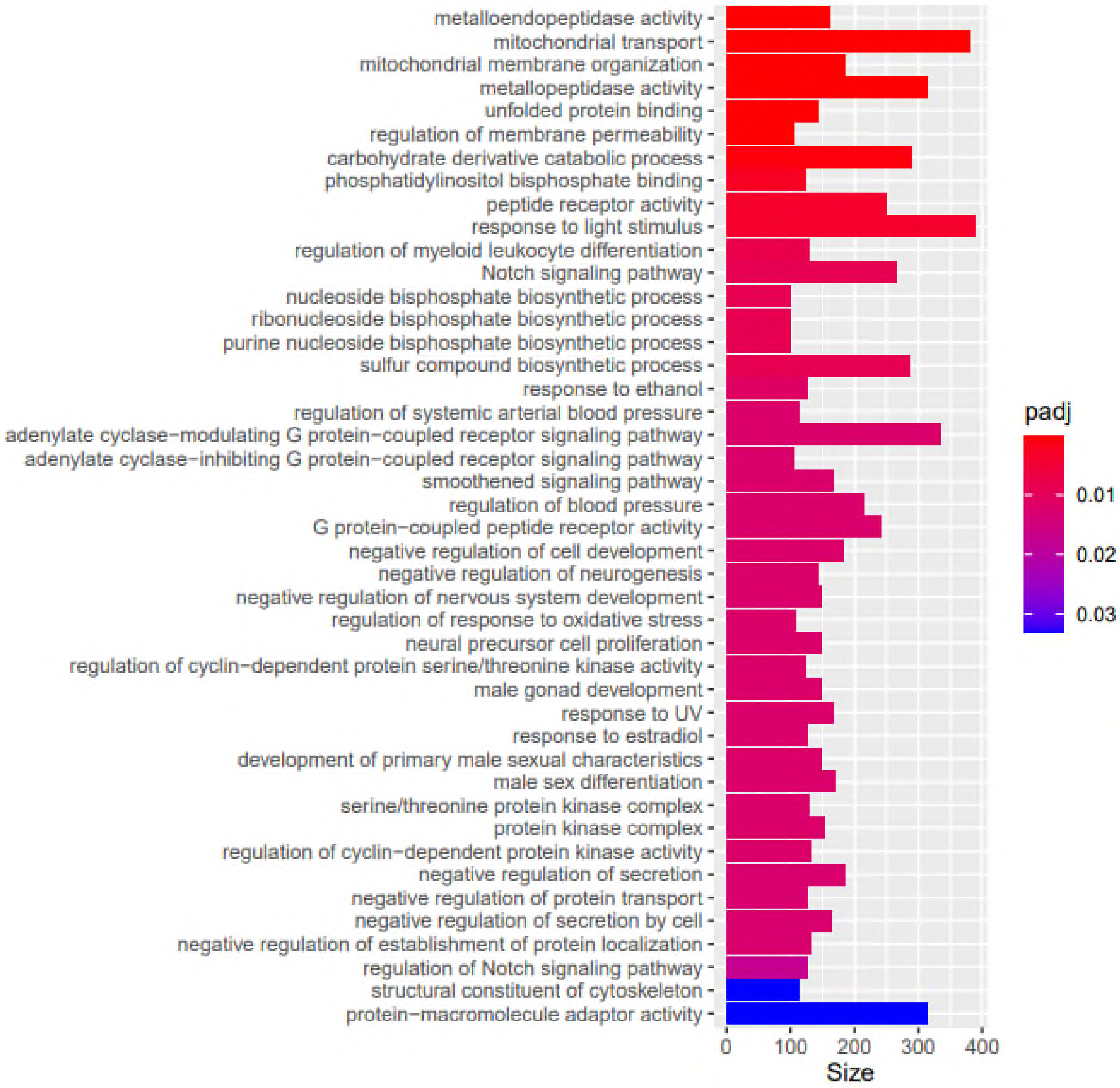

Figure S17: Results of gene ontology (GO) pathway analysis of genes annotated to significant DMPs associated with AD neuropathology in the cortical meta-analysis. Shown for each significant GO category is the pathway size and adjusted P-value.

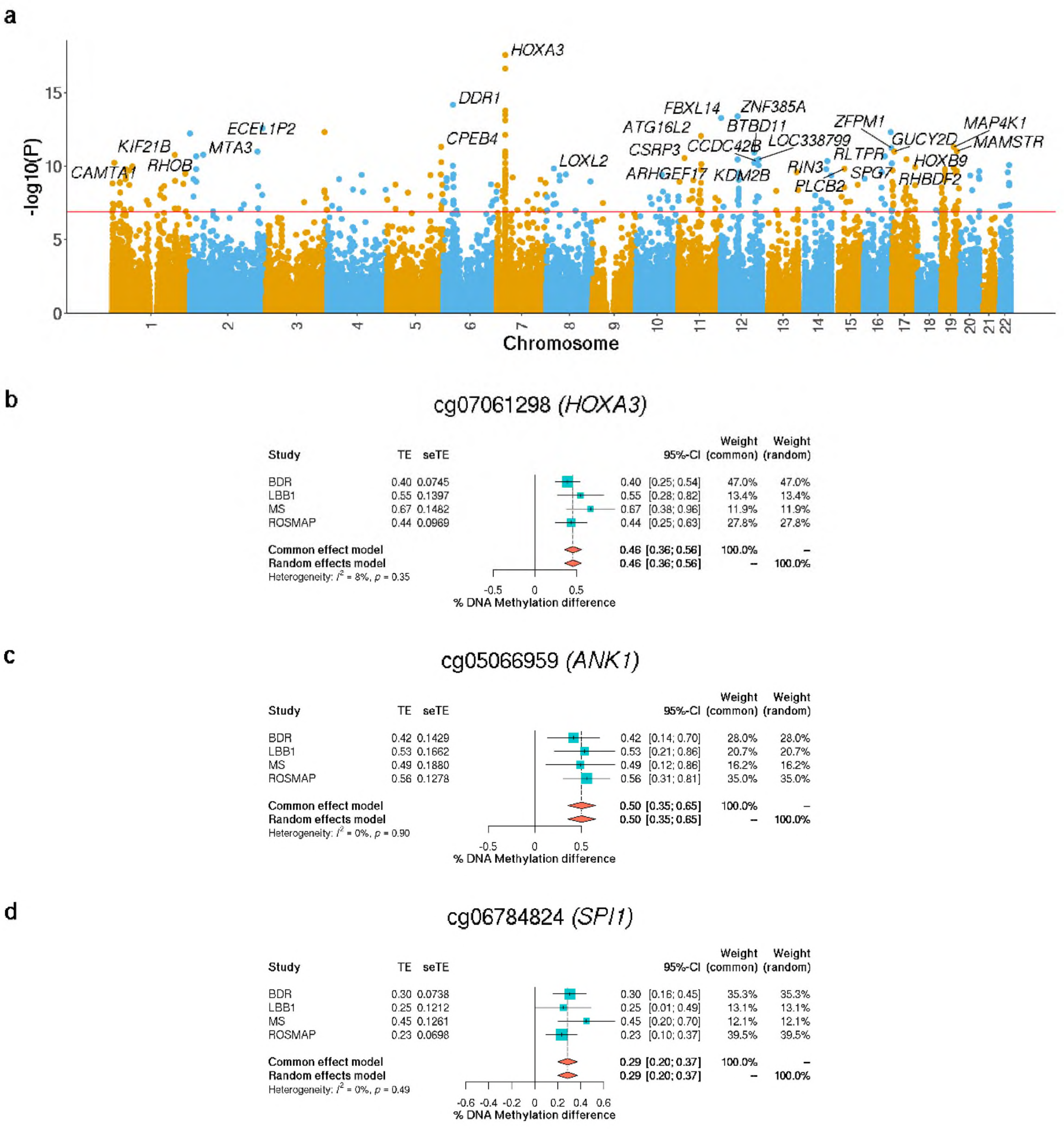

Figure S18: Differentially methylated positions identified in the DLPFC meta-analysis are annotated to genes strongly implicated in Alzheimer's disease. a) Manhattan plot highlighting significant DMPs associated with Braak NFT stage in a DLPFC meta-analysis (N = 1,545). Genes annotated to the 50 most significant DMPs are labelled. The *x*-axis shows chromosomes 1-22 and the *y*-axis shows -log10(P), with the horizontal red line representing the experiment wide significance threshold (P < 9E-8). A complete list of results is given in **Supplementary Data 12. b)** Across all cohorts Braak NFT Stage is associated with hypermethylation at cg22962123 (P = 2.31E-17) which is annotated to *HOXA3*, a gene previously implicated in EWAS of AD[1–3]. **c)** Across all cohorts Braak NFT Stage is associated with hypermethylation at cg05066959 (P = 4.36E-10) which is annotated to *ANK1*, a gene previously implicated in EWAS of AD[1–3]. **d)** Across all cohorts Braak NFT Stage is associated with hypermethylation at cg06784824 (P = 8.97E-10) which is annotated to *SPI1*, a gene previously implicated in GWAS[4,5] and EWAS[1] of AD. The *X*-axis shows the effect size (% DNA methylation difference per SD increase in Braak NFT stage), with squares representing effect size and arms indicating the 95% confidence intervals. Details on each of the cohorts included in the meta-analysis (BDR = Brains for Dementia Research, LBB1 = London 1, MS = Mount Sinai, ROSMAP = Religious Orders Study/Memory and Aging Project) are given in **Supplementary Data 7**.

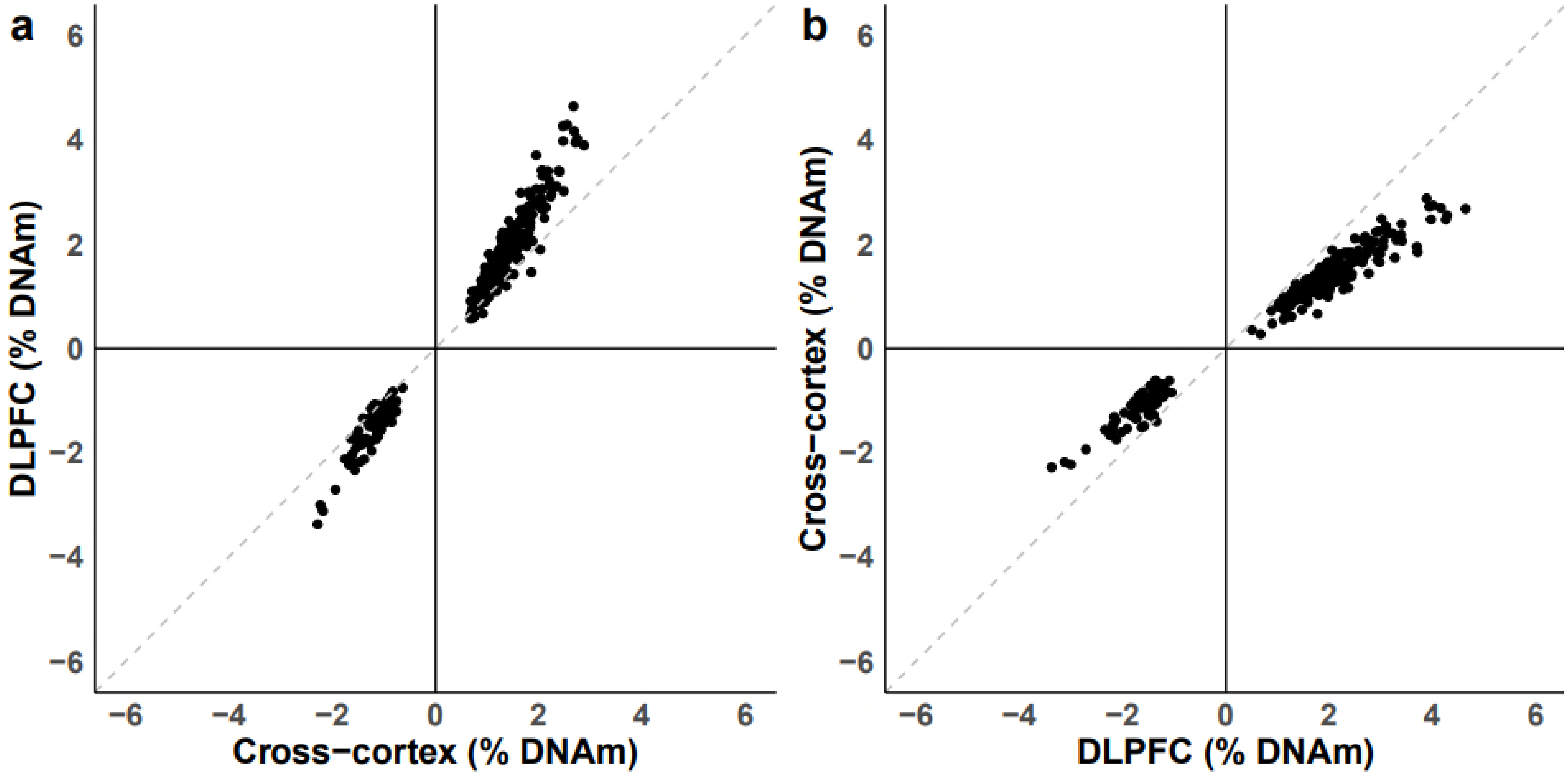

Figure S19: Effects sizes from EWAS meta-analyses of Braak neurofibrillary tangle (NFT) stage undertaken in the DLPFC are highly consistent with those from a cross-cortex meta-analysis. a) Shown are the effect sizes at 334 DMPs identified in the cross-cortex meta-analysis to those same DNA methylation sites in the DLPFC meta-analysis (direction of effect = 100% concordant, sign test P = 2.86E-101). **b)** Shown are the effect sizes at 300 DMPs identified in the DLPFC meta-analysis compared to those same DNA methylation sites in the cross-cortex meta-analysis (concordant = 100%, sign test P = 4.91E-91). The grey dashed line represents y = x.

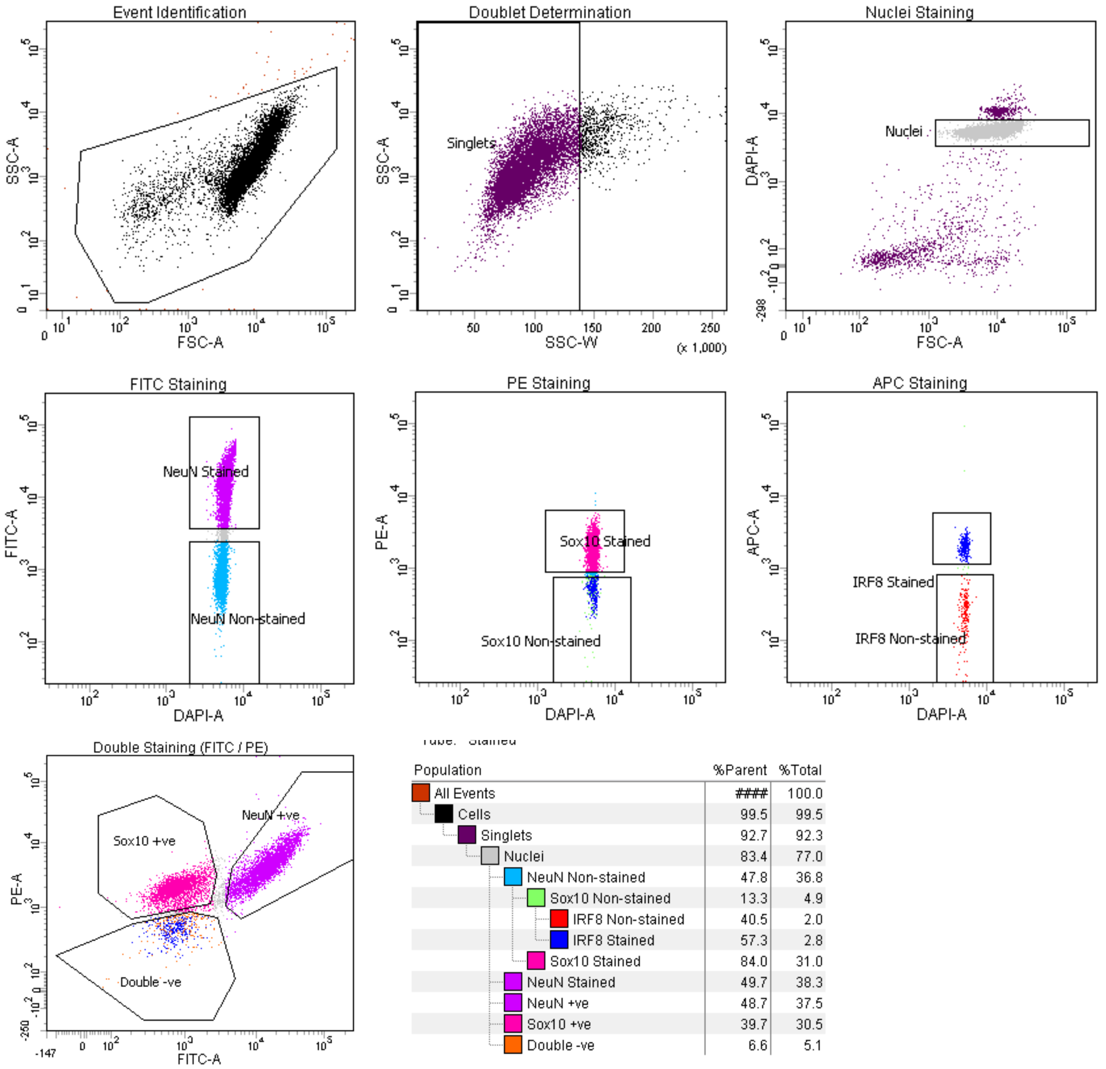

Population	%Parent	%Total
All Events	####	100.0
Cells	99.5	99.5
Singlets	92.7	92.3
Nuclei	83.4	77.0
NeuN Non-stained	47.8	36.8
Sox10 Non-stained	13.3	4.9
IRF8 Non-stained	40.5	2.0
IRF8 Stained	57.3	2.8
Sox10 Stained	84.0	31.0
NeuN Stained	49.7	38.3
NeuN +ve	48.7	37.5
Sox10 +ve	39.7	30.5
Double -ve	6.6	5.1

Figure S20: Isolation of cell-type-enriched nuclei populations from DLPFC tissue using fluorescence activated nuclei sorting (FANS). Shown is the FANS gating strategy for one representative BDR DLPFC sample highlighting the isolation of three discrete nuclei populations (NeuN+, SOX10+, NeuN-/SOX10-) and the overlap of nuclei positive for IRF8, a microglial marker, with the NeuN-/SOX10- population.

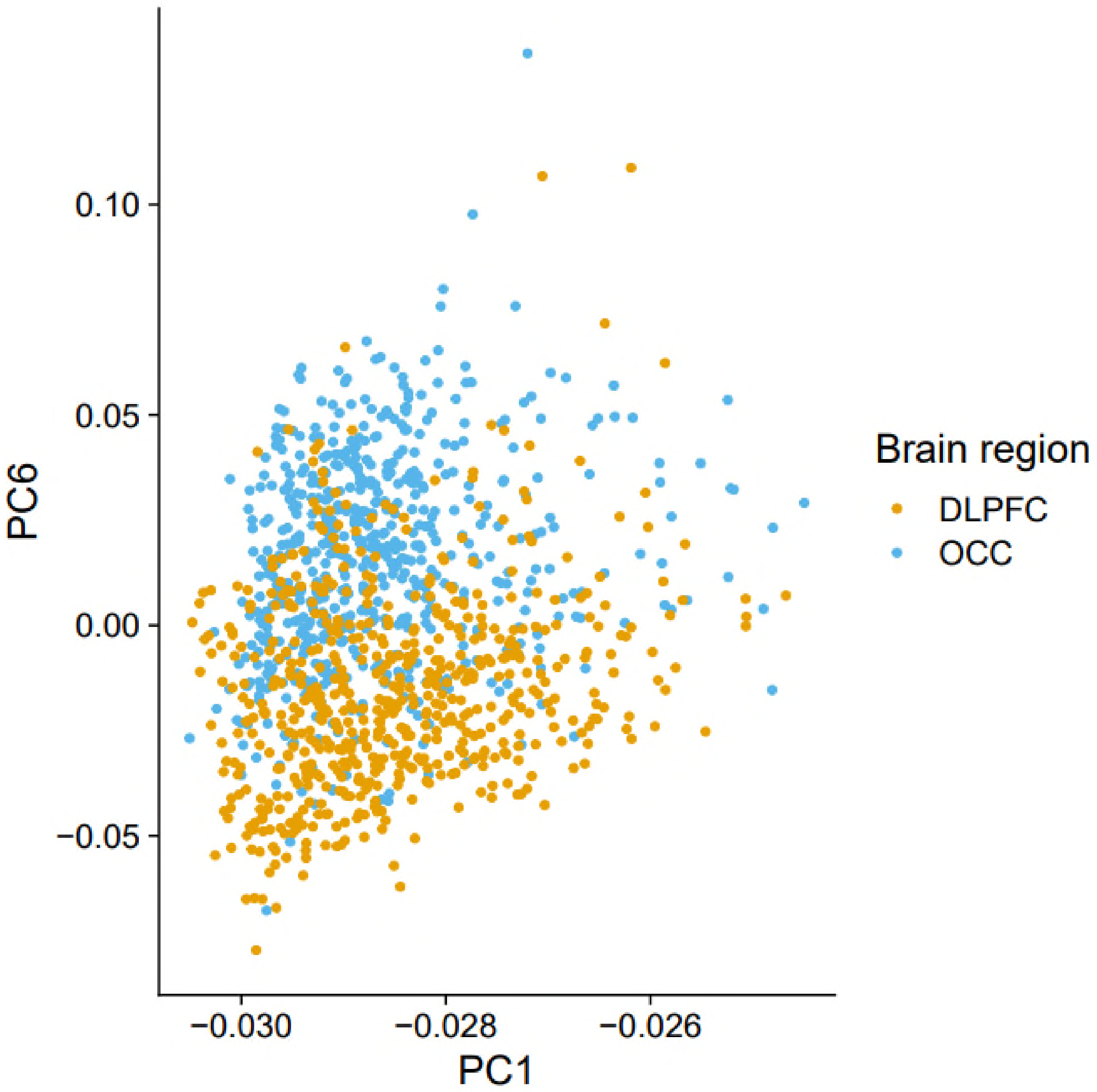

Figure S21: Major principal components of DNA methylation data from both cortical regions largely overlap. PC6 is most associated with brain region (r=-0.54; P = 5.4e-92) but only explains 0.08% of the variance in DNA methylation across samples profiled in this study. Orange = DLPFC (dorsolateral prefrontal cortex [N=610]) and blue = OCC (occipital cortex [N=611]).

Supplementary References

1. Smith, R. G. *et al.* A meta-analysis of epigenome-wide association studies in Alzheimer's disease highlights novel differentially methylated loci across cortex. *Nat. Commun.* **12,** 3517 (2021).
2. Smith, R. G. *et al.* Elevated DNA methylation across a 48-kb region spanning the HOXA gene cluster is associated with Alzheimer's disease neuropathology. *Alzheimers Dement.* **14,** 1580–1588 (2018).
3. Gasparoni, G. *et al.* DNA methylation analysis on purified neurons and glia dissects age and Alzheimer's disease-specific changes in the human cortex. *Epigenetics Chromatin* **11,** 41 (2018).
4. Kunkle, B. W. *et al.* Genetic meta-analysis of diagnosed Alzheimer's disease identifies new risk loci and implicates Aβ, tau, immunity and lipid processing. *Nat. Genet.* **51,** 414–430 (2019).
5. de Rojas, I. *et al.* Common variants in Alzheimer's disease and risk stratification by polygenic risk scores. *Nat. Commun.* **12,** 3417 (2021).
