# Supplementary material for: DNA methylation signatures of Alzheimer’s disease neuropathology in the cortex are primarily driven by variation in non-neuronal cell-types
Source: Nat Commun. 2022 Sep 24;13:5620. doi: 10.1038/s41467-022-33394-7 (PMC9509387; doi:10.1038/s41467-022-33394-7)
Supplement: Supplementary file 3 — Description of Additional Supplementary Files [file 41467_2022_33394_MOESM3_ESM.pdf]

Description of Additional Supplementary Files

Supplementary Data 1: Derived cell proportions are significantly associated with AD pathology measures derived from DNA methylation data in the dorsolateral prefrontal cortex (DLPFC).

Supplementary Data 2: Differentially methylated positions (DMPs) associated with AD neuropathology. In total 67 DMPs were identified at an experiment wide significance threshold (p < 9E-08). Probe information is provided corresponding to chromosomal location (h19/GRCh37 genomic annotation) and the Illumina UCSC gene annotation.

Supplementary Data 3: EWAS results of AD pathology (Braak NFT stage, CERAD score and Thal phase). Summary statistics for the results from four EWAS models using linear regression controlling for major covariates (see **Methods**): 1) EWAS of all 3 neuropathology measures; 2) EWAS of Braak NFT stage; 3) EWAS of CERAD score; and 4) EWAS of Thal phase. Probe information is provided corresponding to chromosomal location (h19/GRCh37 genomic annotation) and the Illumina UCSC gene annotation.

Supplementary Data 4: EWAS of AD pathology conducted in each brain region (prefrontal cortex [DLPFC] and occipital cortex [OCC]) separately. Probe information is provided corresponding to chromosomal location (h19/GRCh37 genomic annotation) and the Illumina UCSC gene annotation.

Supplementary Data 5: Differentially methylated positions (DMPs) associated with 3 AD neuropathology measures (Braak NFT stage, CERAD score and Thal phase) at an experiment wide significance (p < 9e-08) in the dorsolateral prefrontal cortex (DLPFC). Linear regressions were run between DNA methylation and neuropathology at each site. Probe information is provided corresponding to chromosomal location (h19/GRCh37 genomic annotation) and the Illumina UCSC gene annotation.

Supplementary Data 6: Differentially methylated positions (DMPs) associated with 3 AD neuropathology measures (Braak NFT stage, CERAD score and Thal phase) at an experiment wide significance (p < 9e-08) in the occipital cortex (OCC). Linear regressions were run between DNA methylation and neuropathology at each site. Probe information is provided corresponding to chromosomal location (h19/GRCh37 genomic annotation) and the Illumina UCSC gene annotation.

Supplementary Data 7: Cohort characteristics of datasets included in meta-analyses.

Supplementary Data 8: Differentially methylated positions (DMPs) associated with tau pathology in the cross-cortex meta-analysis at Bonferroni significance (P < 1.24E-07). Linear regressions were run between DNA methylation and neuropathology at each site. Probe information is provided corresponding to chromosomal location (h19/GRCh37 genomic annotation) and the Illumina UCSC gene annotation.

Supplementary Data 9: Results from the cross-cortex meta-analysis of Braak NFT stage. Linear regressions were run between DNA methylation and neuropathology at each site. Probe information is provided corresponding to chromosomal location (h19/GRCh37 genomic annotation) and the Illumina UCSC gene annotation.

Supplementary Data 10: Significant gene ontology (GO) categories associated with genes annotated to cross-cortex meta-analysis DMPs. Pathway analysis was conducted using logistic regression controlling for the number of probes in the enrichment analysis.

Supplementary Data 11: Differentially methylated positions (DMPs) associated with tau pathology in the PFC meta-analysis at Bonferroni significance (P < 1.24E-07). Linear regressions were run between DNA methylation and neuropathology at each site. Probe information is provided corresponding to chromosomal location (h19/GRCh37 genomic annotation) and the Illumina UCSC gene annotation.

Supplementary Data 12: Results from the dorsolateral prefrontal cortex meta-analysis of Braak NFT stage. Probe information is provided corresponding to chromosomal location (h19/GRCh37 genomic annotation) and the Illumina UCSC gene annotation.

Supplementary Data 13: Characteristics of the high and low AD pathology FANS sorted samples. Braak high ≥ Braak NFT stage, Braak low ≤ II Braak NFT stage.

Supplementary Data 14: Comparison of effect size for the 334 overlapping tau-associated DMPs identified in our bulk cortex meta-analysis with those at the same sites in an analysis of purified DLPFC nuclei populations from low (Braak NFT stage 0 to II) and high (Braak NFT stage > V) tau-pathology donors.
