# Supplementary material for: DNA methylation signatures of Alzheimer’s disease neuropathology in the cortex are primarily driven by variation in non-neuronal cell-types
Source: Nat Commun. 2022 Sep 24;13:5620. doi: 10.1038/s41467-022-33394-7 (PMC9509387; doi:10.1038/s41467-022-33394-7)
Supplement: Supplementary file 18 — Reporting Summary [file 41467_2022_33394_MOESM18_ESM.pdf]

nature portfolio

Corresponding author(s): JONATHAN MILL - j.mill@exeter.ac.uk

Last updated by author(s): 31-08-2022

Reporting Summary

Nature Portfolio wishes to improve the reproducibility of the work that we publish. This form provides structure for consistency and transparency in reporting. For further information on Nature Portfolio policies, see our Editorial Policies and the Editorial Policy Checklist.

Statistics

For all statistical analyses, confirm that the following items are present in the figure legend, table legend, main text, or Methods section.

n/a	Confirmed	
☐	☒	The exact sample size (*n*) for each experimental group/condition, given as a discrete number and unit of measurement
☐	☒	A statement on whether measurements were taken from distinct samples or whether the same sample was measured repeatedly
☐	☒	The statistical test(s) used AND whether they are one- or two-sided<br>*Only common tests should be described solely by name; describe more complex techniques in the Methods section.*
☐	☒	A description of all covariates tested
☐	☒	A description of any assumptions or corrections, such as tests of normality and adjustment for multiple comparisons
☐	☒	A full description of the statistical parameters including central tendency (e.g. means) or other basic estimates (e.g. regression coefficient) AND variation (e.g. standard deviation) or associated estimates of uncertainty (e.g. confidence intervals)
☐	☒	For null hypothesis testing, the test statistic (e.g. *F*, *t*, *r*) with confidence intervals, effect sizes, degrees of freedom and *P* value noted<br>*Give P values as exact values whenever suitable.*
☒	☐	For Bayesian analysis, information on the choice of priors and Markov chain Monte Carlo settings
☐	☒	For hierarchical and complex designs, identification of the appropriate level for tests and full reporting of outcomes
☐	☒	Estimates of effect sizes (e.g. Cohen's *d*, Pearson's *r*), indicating how they were calculated

Our web collection on statistics for biologists contains articles on many of the points above.

Software and code

Policy information about availability of computer code

Data collection	No software was used for data collection.
Data analysis	Statistical analysis was performed in R 4.1. R Packages used were wateRmelon 1.36.0, bigmelon 1.19.5, meta 5.2.0, lmerTest 3.1.3, lme4 1.1.27, bacon 1.20.0, methylGSA 1.10.0.<br>All scripts for data analyses performed in this manuscript can be found at github.com at https://github.com/gemmashireby/BDR_neuropathology_EWAS.

For manuscripts utilizing custom algorithms or software that are central to the research but not yet described in published literature, software must be made available to editors and reviewers. We strongly encourage code deposition in a community repository (e.g. GitHub). See the Nature Portfolio guidelines for submitting code & software for further information.

Data

Policy information about availability of data

All manuscripts must include a data availability statement. This statement should provide the following information, where applicable:

- Accession codes, unique identifiers, or web links for publicly available datasets
- A description of any restrictions on data availability
- For clinical datasets or third party data, please ensure that the statement adheres to our policy

The BDR DNA methylation data have been deposited in the Dementias Platform UK (DPUK) data portal (https://portal.dementiasplatform.uk/CohortDirectory/Item?fingerPrintID=BDR) and the Gene Expression Omnibus (GEO) at accession number GSE197305 (https://www.ncbi.nlm.nih.gov/geo/query/acc.cgi?acc=GSE197305).

Field-specific reporting

Please select the one below that is the best fit for your research. If you are not sure, read the appropriate sections before making your selection.

- [x] Life sciences
- [ ] Behavioural & social sciences
- [ ] Ecological, evolutionary & environmental sciences

For a reference copy of the document with all sections, see nature.com/documents/nr-reporting-summary-flat.pdf

Life sciences study design

All studies must disclose on these points even when the disclosure is negative.

Sample size	All available BDR samples at the time of our experiments were obtained for DNA methylation profiling, with no pre-selection. Final sample numbers were determined after sample quality control. The BDR bulk tissue dataset cohort consisted of 631 donors. There were a total of 12 donors included in the cell-deconvolution dataset. There were a total of 28 donors included in the analysis of purified nuclei populations. Summary statistics were provided for the remaining meta-analysis cohorts which consisted of an additional 1,408 individuals, representing all available data. Together, this represents the largest study of DNA methylation in Alzheimer's disease yet undertaken.
Data exclusions	Our stringent quality control (QC) pipeline included the following steps: (1) checking methylated and unmethylated signal intensities and excluding poorly performing samples; (2) assessing the chemistry of the experiment by calculating a bisulphite conversion statistic for each sample, excluding samples with a conversion rate <80%; (3) identifying the fully methylated control sample included on each plate was in the correct location; (4) multidimensional scaling of sites on the X and Y chromosomes separately to confirm reported sex; (5) using the 59 SNP probes present on the Illumina EPIC array to confirm that matched samples from the same individual (but different brain regions or nuclei populations) were genetically identical and to check for sample duplications and mismatches; (6) excluding samples with >1% of probes with a detection P value>0.05 and probes with >1% of samples with detection P value >0.05; (8) using principal component (PC) analysis on data from each tissue to exclude outliers based on any of the first three PCs; and (9) the removal of cross-hybridising and SNP probes.
Replication	We investigated if there was concordance in the direction of effect with a previous EWAS of Braak NFT stage and statistically evaluated this using a binomial sign test. We also tested whether DNA methylation differences identified in bulk cortex were replicated in the total nuclei fraction from a subset of donors. In all cases we found very high levels of concordance, as described in the paper.
Randomization	Samples were fully randomized prior to being run on the arrays to avoid bias and technical batch effects.
Blinding	During data QC and pre-processing investigators were blinded to the outcome variables (neuropathology).

Reporting for specific materials, systems and methods

We require information from authors about some types of materials, experimental systems and methods used in many studies. Here, indicate whether each material, system or method listed is relevant to your study. If you are not sure if a list item applies to your research, read the appropriate section before selecting a response.

Materials & experimental systems

n/a	Involved in the study	
☐	☒	Antibodies
☒	☐	Eukaryotic cell lines
☒	☐	Palaeontology and archaeology
☒	☐	Animals and other organisms
☐	☒	Human research participants
☒	☐	Clinical data
☒	☐	Dual use research of concern

Methods

n/a	Involved in the study	
☒	☐	ChIP-seq
☒	☐	Flow cytometry
☒	☐	MRI-based neuroimaging

Antibodies

Antibodies used	Anti-SOX10: pre-conjugated to NL577, supplier: R&D systems, Cat No:NL2864R, dilution: 1:10, clone: 20B7<br>Anti-NeuN: pre-conjugated to Alexa Fluor488, supplier: Millipore, Cat No: MAB377X, dilution: 1:1000, clone: A60<br>Anti-IRF8: pre-conjugated to APC, supplier Invitrogen:, Cat No: 17-9852-82, dilution: 1:150, clone: V3GYWCH
Validation	All antibodies used are pre-conjugated and prevalidated by the manufacturers and extensive work in our lab. Links to specific validation work:<br>SOX10: https://resources.rndsystems.com/pdfs/datasheets/nl2864r.pdf?v=20220831&_ga=2.213932759.1557985042.1661956555-393137340.1661956555<br>NeuN: https://www.merckmillipore.com/GB/en/product/Anti-NeuN-Antibody-clone-A60-Alexa-Fluor488-conjugated,MM_NF-MAB377X#anchor_COA<br>IRF8: https://www.thermofisher.com/order/genome-database/dataSheetPdf?producttype=antibody&pr[...]

tsubtype=antibody_primary&productId=17-9852-82&version=247

Human research participants

Policy information about studies involving human research participants

Population characteristics	Bulk BDR donors: Mean Age (SD) 83.47 (9.11), females=296, males=335, mean Braak NFT stage 3.72 (SD = 1.9), mean Thal Phase 3.09 (SD=1.78), mean LB stage 1.34 (SD =2.26), TDP-43 positive N = 127.<br>Purified nuclei populations: Mean Age (SD) 80.61 (9.09), females=15, males=13, mean Braak NFT stage 3.00 (SD = 2.41), mean Thal Phase 2.23 (SD=1.95), mean LB stage 0.791 (SD =1.72), TDP-43 positive N = 4.<br>Cell-deconvolution reference: Mean Age (SD) 83.17 (11.50), females=5, males=7. All Control/ low pathology.<br>Other meta-analysis datasets:<br>Braak 0-II: Mean Age (SD): 81.47 (7.98), females=163, males=189<br>Braak III-IV: Mean Age (SD): 87.07 (4.99), females=401, males=238<br>Braak V-VI: Mean Age (SD): 84.99 (7.5), females=333, males=200
Recruitment	BDR participants were recruited using both national and local press (e.g. newspapers, newsletters, leaflets), TV and radio coverage as well as at memory clinics and support groups. The remaining meta-analysis cohorts were recruited to relevant brain banks and selected for cohort analysis by original authors.
Ethics oversight	Ethical approval for the study was granted from the University of Exeter Medical School Research Ethics Committee (13/02/009). Data analysis was undertaken using high-performance computing supported by a Medical Research Council (MRC) Clinical Infrastructure award (M008924). The analysis of FANS-purified nuclei was supported by Alzheimer's Research UK (ARUK) grant ARUK-PPG2018A-010 to E.D. DNA methylation data generated in the Brains for Dementia Research cohort was supported by the Alzheimer's Society and Alzheimer's Research UK (ARUK). The BDR is jointly funded by Alzheimer's Research UK (ARUK) and the Alzheimer's Society in association with the Medical Research Council. The South West Dementia Brain Bank is part of the Brains for Dementia Research program, jointly funded by Alzheimer's Research UK (ARUK) and Alzheimer's Society, and is also supported by BRACE (Bristol Research into Alzheimer's and Care of the Elderly) and the Medical Research Council (MRC).

Note that full information on the approval of the study protocol must also be provided in the manuscript.
